# Cellulose-Multiwall Carbon Nanotube Fiber Actuator Behavior in Aqueous and Organic Electrolyte

**DOI:** 10.3390/ma13143213

**Published:** 2020-07-19

**Authors:** Fred Elhi, Anna-Liisa Peikolainen, Rudolf Kiefer, Tarmo Tamm

**Affiliations:** 1 Intelligent Materials and Systems Lab, Institute of Technology, University of Tartu, Nooruse 1, 50411 Tartu, Estonia; elhi.fred@gmail.com (F.E.); anna.liisa.peikolainen@ut.ee (A.-L.P.); tarmo.tamm@ut.ee (T.T.); 2 Faculty of Applied Sciences, Ton Duc Thang University, Ho Chi Minh City 700000, Vietnam

**Keywords:** cellulose-CNT composite fibers, linear actuation, solvent effects, back-relaxation, change of actuation direction

## Abstract

As both consumers and producers are shifting from fossil-derived materials to other, more sustainable approaches, there is a growing interest in bio-origin and biodegradable polymers. In search of bio-degradable electro-mechanically active materials, cellulose-multi wall carbon nanotube (Cell-CNT) composites are a focus for the development of actuators and sensors. In the current study, our aim was to fabricate Cell-CNT composite fibers and study their electro-mechanical response as linear actuators in aqueous and propylene carbonate-based electrolyte solutions. While the response was (expectedly) strongly solvent dependent, the different solvents also revealed unexpected phenomena. Cell-CNT fibers in propylene carbonate revealed a strong back-relaxation process at low frequencies, and also a frequency dependent response direction change (change of actuation direction). Cell-CNT fibers operated in aqueous electrolyte showed response typical to electrochemical capacitors including expansion at discharging with controllable actuation dependence on charge density. While the response was similarly stable in both electrolyte solution systems, the aqueous electrolytes were clearly favorable for Cell-CNT with 3.4 times higher conductivities, 4.3 times higher charge densities and 11 times higher strain.

## 1. Introduction

Polymers are widely used advanced materials, which can be found in almost every object used in our daily life. Since most organic polymers have been derived from non-renewable sources and they accumulate after use in nature, their use has increasingly been found unsustainable. Growing industrial development and diminishing natural resources are creating a demand for technological solutions that utilize renewable materials for multifunctional technologies [1].

Cellulose is the most abundant (organic) polymer available on Earth. As a bio-origin and biodegradable polymer, cellulose is increasingly replacing oil/gas-derived polymers in various applications aiming at sustainability. Due to the strong hydrogen bonding, cellulose is not easily dissolved. Non-derivatizing solvents for cellulose, which do not interact with the hydroxyl groups chemically [2], however, pose environmental hazards hindering their industrial application [3]. These solvents in aqueous complexes include: cuprammonium hydroxide, cupriethylene diamine, CdO/ethylenediamine and NaOH [4]. Cellulose solvents for non-aqueous complexes include: *N*,*N*-dimethylacetamide/LiCl, dimethyl sulfoxide/SO_2_, dimethyl sulfoxide/tetrabutylammonium fluoride. Popular cellulose solvents also include concentrated inorganic salts, mineral acids and N-methylmorpholine oxide [5]. In addition to the environmental problems, these solvents can also have a degrading effect on cellulose [5]. There is a lack of environmentally friendly techniques for dissolving cellulose in popular organic solvents. Negative effects include solvent volatility, toxicity, formation of toxic gases, difficulty in solvent regeneration, the amount of dangerous or unusable byproducts and the low solubility of cellulose [6,7]; these can be evaded by dissolving cellulose directly in ionic liquids (IL) [8]. When dissolving cellulose in IL, the inter- and intramolecular hydrogen bonds in cellulose break down as new hydrogen bonds between cellulose and the dissolving IL [9] are formed. Many ILs can break the hydrogen bonded network of cellulose without cellulose derivatization [10].

A number of applications for cellulose composites have been proposed, such as supercapacitors [11], electrode material for Li-batteries [12] and multifunctional materials [13]. Cellulose-based composites with carbon nanotubes [14,15,16], reduced graphene oxide [17], conducting polymers [18] and others have come in focus for environmentally sustainable solutions, providing opportunities to form materials with usable electrical conductivity and switchable electrical and optical properties due to the wide range of morphological forms of cellulose [19].

Cellulose-based materials have previously been proposed to be attractive for environmentally sustainable electromechanical actuators because of their low-voltage operation as well as biodegradability. Previous research [20] has introduced bending tri-layer actuators based on IL-cellulose solutions mixed with multi-walled carbon nanotubes (MWCNTs) forming the electrodes on both sides of a chitosan separator. Applied voltage up to 5 V led to a bending displacement. Applying bio-renewable materials for already existing applications, e.g., linear polymer actuators, has become a major objective in the actuator field.

Carbon nanotube (CNT) was chosen as the conducting component in the present research for its mechanical strength, chemical and mechanical stability, as well as conductivity. So far, no linear actuation studies using cellulose-MWCNT composites (Cell-CNT) fibers have been presented. Our main focus of the study apart from showing it is possible was the interplay with solvent as we applied the same salt LiTFSI in aqueous and propylene carbonate-based electrolytes to discuss any changes of actuation response. The main hypotheses the present research sought to confirm were the following: it is possible to fabricate an electromechanically responsive composite material based on cellulose and CNT; due to the nature of cellulose, the obtained composite would show increased response stability compared to other electro(chemo)mechanically responsive polymeric materials such as conducting polymers, while the response would be strongly solvent dependent due to the hydrophilic nature of cellulose.

The electroactivity (charging) of the material studied in this research was mostly interpreted as a non-faradaic process where the formation of an electrical double layer (EDL) was induced by charge injections [13]. As the Cell-CNT is a composite, the interactions between ions from the cellulose network, solution and CNT need to be considered. A set of electro-chemo-mechanical deformation (ECMD) measurements, driven by cyclic voltammetry at different scan rates and square wave potential steps at different applied frequencies was carried out. The materials were characterized using scanning electron microscopy (SEM), energy dispersive X-Ray (EDX) and Fourier-transform infrared spectroscopy (FTIR).

## 2. Experimental

### 2.1. Materials

Commercial microcrystalline cellulose (average particle size of 20 μm), 1-ethyl-3-methylimidazolium chloride ((EMIM-Cl), >97%), bis(trifluoromethane)sulfonimide lithium salt (LiTFSI, 99.95%) and propylene carbonate (PC, 99%) were purchased from Sigma-Aldrich (Taufkirchen, Germany) and used as received. Multi-walled carbon nanotubes (Baytubes ^®^ C 150 P; amorphous carbon content 0%, average outside diameter 13 nm, average inside diameter 4 nm, length >1 µm; Bayer Material Science, Leverkusen, Germany) were used as received. Deionized water (Milli-Q+, Tallinn, Estonia) was applied for aqueous solutions.

### 2.2. Formation of Cell-CNT Composite Fibers

Cellulose (at 10 wt% concentration) was dissolved in EMIM-Cl by heating the solution at 85 °C for 12 h and mechanically stirring every 6 h. After this, a suspension of 10 wt% of MWCNT in EMIM-Cl was added to the cellulose solution and mixed [8]. The cellulose solution with dispersed nanotubes was ultrasonically dispersed for 10 min (Hielscher UP200S, 200 W, 24 kHz, Mount Holly, NJ, USA), and immediately extruded through a syringe (inner diameter 0.76 mm) forming a cylindrical filament. The cellulose solution with dispersed nanotubes was extruded through a syringe (inner diameter 0.76 mm), forming a cylindrical filament. Deionized water was used as the anti-solvent to obtain composite fibers after regeneration for 5 min. The anti-solvent was used in overabundance (ca 100 mL of water per 1 g of EMIM-Cl) to remove EMIM-Cl from the composite structure, and to regenerate cellulose. After removing the regenerated composite from water, it was left to dry in open air at room temperature. The cellulose-MWCNT fibers (Cell-CNT) had a diameter of 0.88 ± 0.05 mm. Three different patches of fibers were produced in similar manner and their properties were studied.

### 2.3. Linear Actuation Measurements of Cell-CNT Composite Fibers

The dried Cell-CNT fibers were cut in 2 cm length pieces and soaked in electrolyte solution for 24 h. The strain ε (ε (%) = (L_1_ − L_0_)/L_0_ × 100 (constant force of 0.5 mN)) and stress σ (σ = weight (g) × g (9.81 m s^−2^)/(fiber volume/π × r^2^)) of Cell-CNT fibers were measured in a three electrode cell (Ag/AgCl (3M KCl) reference electrode, platinum sheet counter electrode) with the fixed samples as the working electrode between a force sensor (TRI202PAD, Panlab, Barcelona, Spain) and gold contact clamp operated with an in-house software [21] with the linear muscle analyzer setup.

The Cell-CNT fibers were operated in 0.1 M LiTFSI aqueous (LiTFSI-aq) or propylene carbonate-based (LiTFSI-PC) solution using cyclic voltammetry (potentiostat, Biologic PG581, Göttingen, Germany) at varied scan rates (5 mV s^−1^, 20 mV s^−1^, 50 mV s^−1^ and 100 mV s^−1^) and square wave potential steps (chronopotentiometry) at selected frequencies from 0.0025 Hz to 0.1 Hz. Applying Equations (1) and (2), the diffusion coefficients (D) were determined from the chronopotentiometric response [22].

(1) $$\ln\left[1-\frac{Q}{Q_{t}}\right]=-bt$$

(2) $$D=\frac{b\cdot h^{2}}{2}$$

The diffusion coefficients were obtained by applying Equations (1) and (2). The current density time curves at each frequency are integrated obtaining the charge density at each time (Q) divided by the total charge density Q_t_. If the left side of Equation (1) is plotted against the time, the slope b is obtained. The thickness of the Cell-CNT samples (h) with the obtained slope b led to the diffusion coefficients at oxidation and reduction (Equation (2)).

### 2.4. Material Characterization

The Cell-CNT samples were analyzed by scanning electron microscopy (Helios NanoLab 600, FEI, Hillsboro, OR, USA) and EDX spectroscopy (Oxford Instruments with X-Max 50 mm^2^ detector, Concord, MA, USA). The Cell-CNT samples were, after actuation measurements, charged (0.55 V, 5 min), cut, discharged (−0.8 V, 5 min) and the pieces dried and EDX spectroscopy made from cross-section images. The molecular structure of the Cell-CNT fibers in the oxidized state (0.55 V) was examined with Fourier transform infrared spectroscopy (FTIR) (4000–500 cm^−1^, 24 scans, Bruker Alpha with Platinum ATR, Billerica, MA, USA). Resistivity of the samples was measured with a digital multi-meter (LCR Meter LCR200, EXTECH Instruments, Taipei, Taiwan) and the electric conductivity σ_e_ was obtained (R is the surface resistivity and ω the thickness of the Cell-CNT fibers) following Equation (3).

(3) $$\sigma_{e}=\frac{1}{\left(R\times \omega \right)}$$

## 3. Results and Discussion

**Scheme 1:**
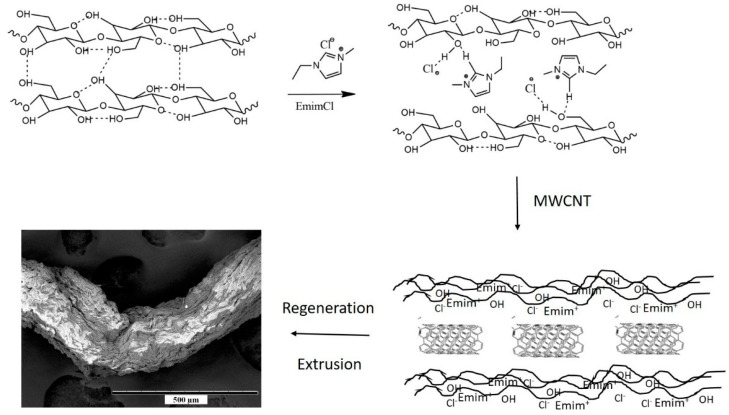
Fabrication of cellulose-multi wall carbon nanotube (Cell-CNT) fibers with cellulose dissolution in 1-ethyl-3-methylimidazolium chloride (EMIM-Cl) inclusion of multi-walled carbon nanotubes (MWCNT), extrusion of the mixture and regeneration to form the fibers (scanning electron microscopy (SEM) images, scale bar 500 µm).

Fibers can be considered the preferred form of composite materials for the envisaged applications in wearable and smart fabrics. The Cell-CNT composites fabricated in this work were made and characterized in a multi-step process. Dissolving cellulose in ionic liquids was done adhering to a standard protocol [5]; the process of making Cell-CNT fibers is shown in Scheme 1. The capability of IL to dissolve cellulose relies on the strong ionic interactions of the IL ions. More specifically, it is understood that the anions bind the hydroxyl groups of the anhydroglucose unit, breaking the hydrogen bonds and allowing the separation of the chains, leading to the dissolution of cellulose [9]. Ionic liquids that contain non-coordinating anions, e.g., [BF_4_]^−^ and [PF_6_]^−^, are non-solvents for cellulose [9,14]. EMIM-Cl was chosen in this work for its effectiveness in dissolving cellulose [5,23].

After solubilizing cellulose and mixing it with MWCNT, the IL can be recycled almost without loss after the regeneration of the fiber in water which acted as the anti-solvent. Most common anti-solvents for this purpose are water, ethanol, acetone, methanol and acetonitrile [24]. The anti-solvent breaks the coordination between IL and cellulose, and hydrogen bonds (inter- and intramolecular) between cellulose molecules are reformed, debilitating the solubility of cellulose, and thus, regenerating the cellulose. Dynamic analysis shows that in the case of acetate anions in water, the anions have the shortest opposition time near cellulose and the highest agility compared to acetate anions in ethanol and acetone. This makes water the preferred anti-solvent. It outperforms ethanol and acetone due to its higher binding energy with ionic liquids and mobility. Water is the most popular anti-solvent for regenerating cellulose in non-simulation experiments; it also outperforms methanol when regenerating wood from a wood/ionic liquid mixture [25].

Water was chosen to be the anti-solvent used in this work, as it regenerates the dissolved cellulose at the fastest rate [10] and to the purest form, forming Cell-CNT fibers (Scheme 1) over extrusion through a syringe, when compared to other anti-solvents available in the literature. Already a 1% content of water in IL-cellulose solution weakens the coordination of IL and cellulose, rendering the cellulose insoluble [10,26].

### 3.1. Characterization of Cell-CNT Fibers

**Figure 1:**
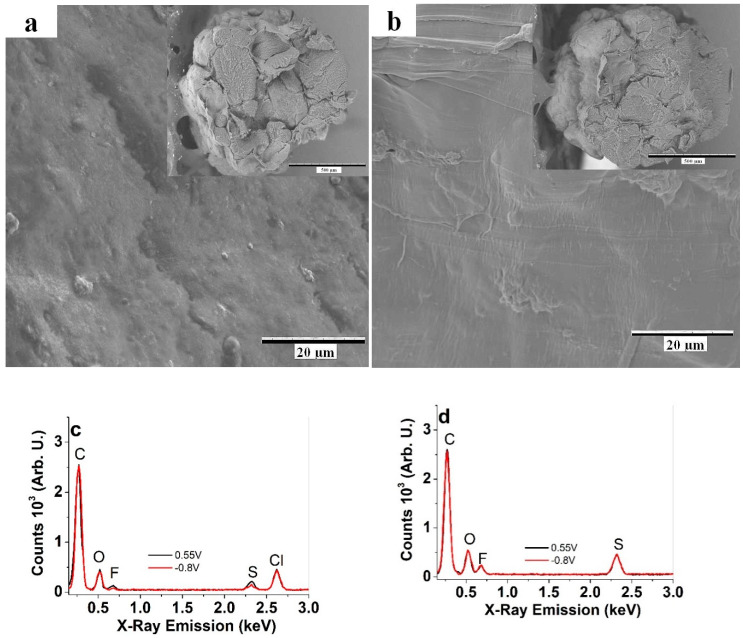
SEM images of the surface (scale bar 20 µm) and cross section (inset, scale bar 500 µm) of cellulose-multi wall carbon nanotube (Cell-CNT) fibers operated (200 actuation cycles) in (**a**) bis(trifluoromethane)sulfonimide lithium in propylene carbonate (LiTFSI-PC) and (**b**) LiTFSI in aqueous solvent (LiTFSI-aq). Energy dispersive X-Ray (EDX) spectra of Cell-CNT fibers in charged (0.55 V, black line) and discharged (−0.8 V, red line) state are presented in (**c**) for LiTFSI-PC and (**d**) for LiTFSI-aq electrolytes. (Li^+^ ions are not detectable in EDX spectroscopy).

SEM images of the surface and cross section (scale bar 500 µm) of Cell-CNT cycled in LiTFSI-PC and LiTFSI-aq are introduced in Figure 1a,b and the corresponding EDX spectra of the cross-sectional images are shown in Figure 1c,d.

The SEM surface images of the Cell-CNT fibers operated in LiTFSI-PC (Figure 1a) reveal a rougher surface than those operated in LiTFSI-aq (Figure 1b). It can be noticed that the Cell-CNT fibers swell in aqueous LiTFSI in a range of 14% resulting in a diameter of 0.92 ± 0.08 mm while in LiTFSI-PC a swelling rate of 8% is found, giving a diameter of 0.88 ± 0.07 µm. The electric conductivity of the Cell-CNT fibers right after formation is 0.2 ± 0.02 mS cm^−1^, and stays at 0.22 ± 0.02 mS cm^−1^ after actuation in LiTFSI-PC. In LiTFSI-aq the electrical conductivity increases to 0.75 ± 0.05 mS cm^−1^. To evaluate the ion content after actuation, EDX spectroscopy was performed at charged (5 min, 0.55 V) and discharged (5 min, −0.8 V) states, shown in Figure 1c,d with a strong carbon (C) peak at 0.26 keV, an oxygen peak (O) at 0.52 keV, a fluorine peak (F) at 0.68 keV, a sulfur peak at 2.32 keV and a chlorine peak (Cl) at 2.62 keV. If operating in LiTFSI-PC, the fluoride, sulfur and partly oxygen peaks belonged to [TFSI]^-^ ions. Their intensity reduced slightly on discharging in comparison to the charged state. Interestingly, a strong Cl peak was observed (Figure 1c), which in all likelihood belongs to the chloride ions from EMIM-Cl staying in the Cell-CNT fibers, even after a long regeneration time. In the case of LiTFSI-aq electrolyte (Figure 1d) the Cl peak disappeared while the intensity of the other peaks (C, O, F and S) did not change during charging/discharging cycles. Apparently, the larger swelling of cellulose in water together with higher solubility allows the chloride ions to be washed out (or exchanged) during cycling.

**Figure 2:**
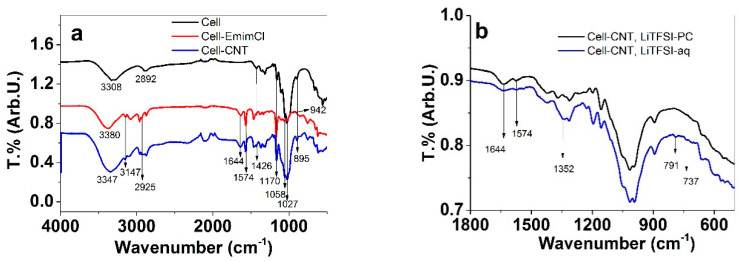
Fourier-transform infrared spectroscopy (FTIR) spectra: (**a**) unprocessed cellulose (black line), cellulose with 1-ethyl-3-methylimidazolium chloride (EMIM-Cl) (red line) and regenerated cellulose-MWCNT composite (blue line); (**b**) the comparison of Cell-CNT actuated in LiTFSI-PC (black line) and LiTFSI-aq (blue line).

FTIR spectra of the unprocessed cellulose, cellulose regenerated after dissolving in EMIM-Cl and Cell-CNT composites are presented in Figure 2a. The spectra of Cell-CNT fiber actuated in LiTFSI aqueous and propylene carbonate solutions in the oxidized state at 0.55 V are shown in Figure 2b.

In the FTIR spectra, the peaks at 3306 cm^−1^ represent the stretching vibration of OH bonds [27], which are shifted to 3380 cm^−1^ in the case of cell-EMIM-Cl and 3347 cm^−1^ in the case of Cell-CNT. The main reason for the shifts is related to changes of inter- and intramolecular hydrogen bonds (Scheme 1) due to the addition of EMIM-Cl and dissolution. The peaks at 2925 cm^−1^, 2992 cm^−1^ and 3147 cm^−1^ are related to –C–H bond vibrations. Typical cellulose peaks [27] are also found at 1426 cm^−1^ corresponding to the C–H stretching vibrations of CH_2_ groups, at 1170 cm^−1^ caused by asymmetric stretching vibrations of the C–O–C bridge, at 1058 cm^−1^ corresponding to bending of C–H bonds, at 1027 cm^−1^ for the skeleton vibration of C–C bonds and at 897 cm^−1^ where the peak (shifted to 942 cm^−1^ in cell-EMIM-Cl) represents C–H bond vibrations [14]. In Cell-EMIM-Cl and Cell-CNT, several peaks representing the EMIM-Cl units (Scheme 1) were found, characteristic of skeleton vibration of the imidazolium ring with peaks at 1644 cm^−1^ and 1574 cm^−1^ [28]. It can be concluded that the EMIM^+^ cations were present after dissolving cellulose and remained (at least partially) present in regenerated Cell-CNT fibers. Figure 2b represents the Cell-CNT samples operated in LiTFSI-PC and LiTFSI-aq electrolytes. The characteristic peaks of the imidazolium ring vibrations at 1644 cm^−1^ and 1574 cm^−1^ were still found, although with lower intensity in the case of LiTFSI-aq. Additional peaks for Cell-CNT in LiTFSI-aq revealed [TFSI]^-^ inclusion [29]: the peak at 1352 cm^−1^ represents the asymmetric SO_2_ stretching mode, small peaks at 791 cm^−1^ (788 cm^−1^ from the literature [29]) show the CF_3_ symmetric bending mode and the band at 737 cm^−1^ corresponds to S-N stretching mode. The increased fluorine peak in the EDX spectrum in Figure 1d reveals a higher amount of TFSI^-^ incorporated during charging/discharging of Cell-CNT if operated in LiTFSI-aq electrolyte.

### 3.2. Linear Actuation of Cell-CNT Fibers

The embedded MWCNT in cellulose determines the electroactivity and the electromechanical response of the composite. To investigate the linear actuation properties of Cell-CNT strain and stress, measurements under cyclic voltammetric and chronopotentiometric driving were performed. While electromechanically active in both solvents, the responses of Cell-CNT fibers in aqueous and organic solutions were rather different.

#### 3.2.1. Cyclic Voltammetry

**Figure 3:**
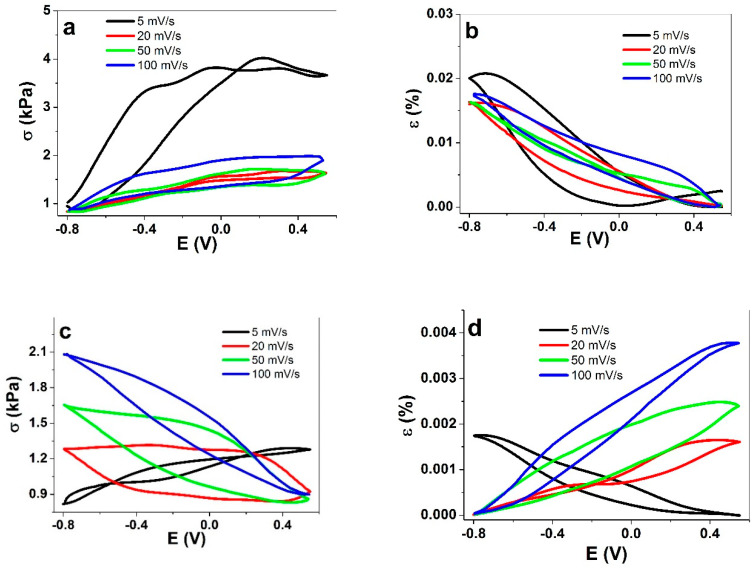
Cyclic voltammetric measurements (3rd cycles) of Cell-CNT fibers (the working electrode) at different scan rates (5 mV s^−1^ (black); 20 mV s^−1^ (red); 50 mV s^−1^ (green) and 100 mV s^−1^ (blue)) operated in a three-electrode setup with an Ag/AgCl (3M KCl) reference electrode and a platinum sheet counter electrode. The stress of Cell-CNT in LITFSI-aq is shown in (**a**) and the strain in (**b**). Cell-CNT in LiTFSI-PC electrolyte presents stress in (**c**) and strain in (**d**) against the applied potential range from 0.55 V to −0.8 V.

The linear actuation response of the Cell-CNT fibers performed in LiTFSI-aq and LiTFSI-PC driven by cyclic voltammetry (scan rate 5 mV s^−1^, 20 mV s^−1^, 50 mV s^−1^ and 100 mV s^−1^) are summarized in Figure 3a–d. The corresponding current densities and charge density curves are shown in Figure S1a–d.

The linear actuation response of Cell-CNT in LiTFSI-aq electrolyte (Figure 3a,b) reveals that indifferent of the scan rate, the main expansion at discharging took place, reaching, at the lowest scan rate of 5 mV s^−1^, 0.02% of strain (stress 2.7 kPa, the stress is always opposite to strain). Figure 3c,b reveals that at a scan rate of 5 mV s^−1^ an expansion at discharging takes place. At higher scan rates, the actuation direction changes to expansion upon charging. In absolute terms, both stress and strain first decrease, then increase with increasing scan rate: stress from 0.45 kPa at 5 mV s^−1^ (expansion at discharging), to 1.14 kPa at 100 mV s^−1^ (mainly expansion at charging). The strain values were rather low, reaching just 0.004% at 100 mV s^−1^. Nanostructured carbon materials, like MWCNT fibers in LiTFSI-PC [30] follow the EDL process with main expansion at discharging. It was found that the charging/discharging mechanism of MWCNT materials cannot be attributed to one single process as it can involve EDL (electrostatic double layer) effects [31], quantum mechanically induced C-C length change [31] and also faradaic processes [32]. In case of Cell-CNT composites, the electrical conductivity is significantly lower due to the non-conductive cellulose chains wrapped around CNT, but also due to the different ion content and mobility inside the composite. The existence of both anions and cations from EMIM-Cl, which we assume appeared as separate entities in Cell-CNT, were evident as well during charging/discharging cycles in LiTFSI-PC at low scan rates (Figure 3c,d). The expansion at discharging was attributed to the injection of Li^+^ ions to balance the charge of some anions that remained entrapped inside Cell-CNT, such as chloride from the ionic liquid and perhaps a small amount of [TFSI]^−^. At faster scan rates the process was increasingly reversed, with expansion upon charging.

The main reason for vastly improved linear actuation in water as compared to PC was likely the higher conductivity of Cell-CNT in LiTFSI-aq as well as the hydrophilic property of cellulose, which led to a much higher swelling rate of the composite in aqueous solutions than in propylene carbonate. The latter is also supported by the EDX results (Figure 1d), with the absence of the chloride peak. FTIR spectroscopy (Figure 2b) shows reduced imidazolium peaks for Cell-CNT operating in LiTFSI-aq electrolyte. Taking into consideration that some of the EMIM-Cl is removed with mostly all chloride expelled, the observation of [TFSI]^-^ anions in EDX (Figure 1d, fluoride and sulfur peak) and FTIR (Figure 2b) suggest that [TFSI]^-^ replaced the chloride in cellulose. Taking into account that EDX showed that the [TFSI]^-^ content does not change during the charging/discharging process, it has to be assumed that cation (Li^+^) flux from LiTFSI-aq solution induces the volume changes upon discharging, compensating for the poorly mobile residual charged species. The complete mechanism and diverse processes of Cell-CNT composite fiber linear actuation need further investigation.

The charge densities (Figure S1a) show that in the LiTFSI-aq solution the charging/discharging is in balance with the highest charge density of 124 mC cm^−2^ found at scan rate 5 mV s^−1^, and decreasing with increased scan rates as expected (Figure S1b). Due to the high ohmic resistance in LiTFSI-PC the Cell-CNT fiber reveals at the scan rate of 5 mV s^−1^ (Figure S1c) an atypical capacitor shape. The charging/discharging curves in Figure S1d show a charge density of 29 mC cm^−2^ at a scan rate of 5 mV s^−1^, decreasing with increased scan rate to 5.6 mC cm^−2^ at 100 mV s^−1^.

It can be concluded that the solvent of the electrolyte has a major role in the Cell-CNT electrochemical response and actuation. The high swelling of cellulose in water was applied in recent research, applying a cellulose MWCNT composite to sense water [33], while with similar composites formed with an aerogel, volatile organic vapor sensing was obtained [34].

The hydrophilic Cell-CNT polymer is much more suitable for application in aqueous solutions, as indicated by a charge density 4.3 times higher, a strain 11 times higher, electronic conductivity 3.4 times higher and overall a much more consistent response, as compared to propylene carbonate-based solutions.

#### 3.2.2. Square Wave Potential Step Measurements

**Figure 4:**
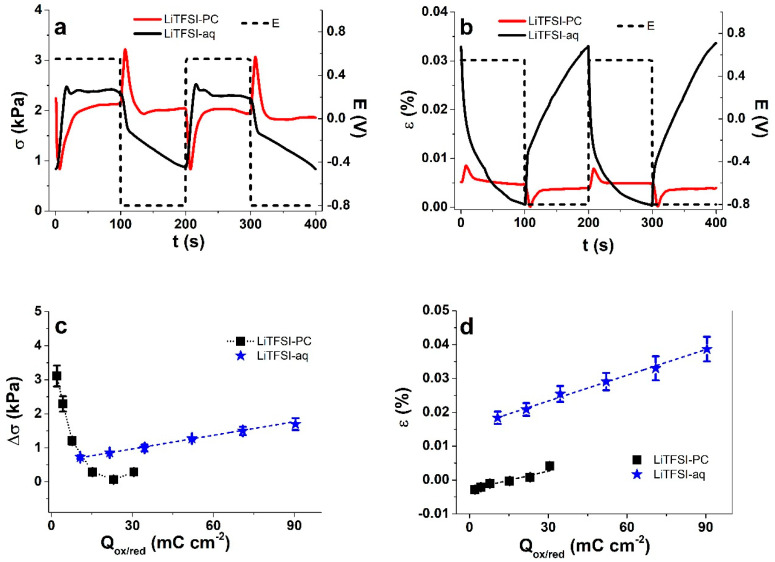
(**a**) Stress and (**b**) strain of Cell-CNT fibers in square wave potential step measurements in LiTFSI-PC (red, dotted) and LiTFSI-aq (black, solid) with equal LiTFSI concentration. Presented are two subsequent cycles (2nd and 3rd) at 0.005 Hz. Charge densities Q of Cell-CNT fibers in LiTFSI-PC (■) and LiTFSI-aq (★) at different applied frequencies (shown in Figure S2a,b) against (**c**) stress difference Δσ and (**d**) strain ε (negative values of strain indicate expansion at charging, positive values show expansion at discharging). The linear fit (dashed blue) of Cell-CNT in LiTFSI-aq (**c**,**d**) are shown for orientation only.

To evaluate the Cell-CNT linear actuation in LiTFSI-PC and LiTFSI-aq electrolytes under abrupt polarization changes, square wave potential step measurements were performed. The profiles of stress and strain of Cell-CNT at the frequency 0.005 Hz are presented in Figure 4a,b, respectively. The charge density was determined from current density time curves (the curve at 0.005 Hz is shown in Figure S2) and the results for stress and strain are presented in Figure 4c,d. The dependency of stress and strain on frequency is shown in Figure S3a,b.

Figure 4a,b compare the stress and strain response of Cell-CNT operated in LiTFSI in aqueous and PC solutions in two subsequent cycles driven by square wave potential steps (0.005 Hz). Other frequencies can be seen in Figure S4. The stress and strain curves in both solvents were markedly different, and especially in the case of LiTFSI-PC for which the behavior is rather complicated, with fast expansion/contraction processes upon both charging and discharging. The EDL formation mechanism cannot easily explain this rather complicated response, as due to mixed ion participation the net stress and strain were reduced, accompanying the restructuring of the double layer. The response observed here represents back relaxation processes where the initially increasing stress upon discharging was followed by rapid decrease, with the opposite (but similar magnitude) seen upon charging. The back relaxation process often occurs in actuators with carbon-based electrodes (like carbide-derived carbon) [35] due to viscoelastic effects [36]. By 0.1 Hz, the back relaxation subdues, therefore, the frequency plays a key role in the observed response, shown as well in other low voltage operated ionic electroactive polymers [37]. The solvent also clearly plays an important role, as the response direction is the opposite for water and PC. While a small back relaxation effect can also be observed in case of stress upon of charging in an aqueous solution, it is neither visible on the discharging leg nor on the strain curve. As cations are typically more solvated due to higher charge density, the stronger swelling in water allows cations to be more mobile in the case of aqueous solution. In PC, the limited flexibility of cellulose fibers together with low swelling [38] limit cation transport. The correlation between charge density and stress and strain are shown in Figure 4c,d. While in LiTFSI-aq the relations have a strong linear character (electrochemical capacitor-like behavior), the relation in LiTFSI-PC is clearly nonlinear, especially for stress. At low charge density—where much lower driving force is applied to ions—the stress values reached in LiTFSI-PC were significantly higher (3 kPa vs. 0.7 kPa) than in LiTFSI-aq at the same frequency. While the absolute value of the obtained strain peaking at 0.04% for Cell-CNT in LiTFSI-aq electrolyte solution can be considered rather low, other composites of cellulose with electroactive electrode materials such as polypyrrole deposited on carbon coated cellulose [39] have shown strains in a similar range of 0.06%. However, the MWCNT embedded inside cellulose over the top-coated conducting polymers has several advantages, such as mechanical durability and chemical stability, especially against overoxidation.

**Figure 5:**
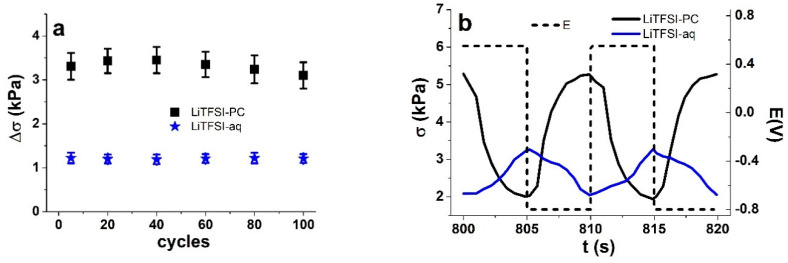
Long-term measurements of square wave potential steps at 0.1 Hz of Cell-CNT fibers showing in (**a**) the stress difference Δσ against cycle number in LiTFSI-PC (■) and LiTFSI-aq (★). Cycles 80-81 of Cell-CNT fibers showing stress σ against time t and in (**b**) of LiTFSI-PC (black line) and LiTFSI-aq (blue line) electrolytes with applied potential E (dashed line) from 0.55 V to −0.8 V.

To investigate the durability and stability of Cell-CNT fibers, square wave potential step measurements at 0.1 Hz frequency were performed for 100 cycles; Figure 5a and Figure S5 show the stress difference Δσ evolution in LiTFSI-PC and LiTFSI-aq. Figure 5b compares the stress response curves (cycles 80-81) of Cell-CNT fibers in the two solutions.

The stress differences of Cell-CNT fibers operated in LiTFSI-PC show a slight increase (from 3.3 kPa to 3.45 kPa) by cycle 40, to a decrease to 3.1 kPa by cycle 100 (Figure 5a). In LiTFSI-aq, the stress differences have an approximately constant response of 1.21 kPa throughout the 100 cycles. After making 80 cycles (Figure 5b), the response of Cell-CNT fibers still follows the same logic as initially (Figure S4), with stress increasing upon charging for LiTFSI-aq and decreasing in LiTFSI-PC. Therefore, as the stability appears reasonable (in PC) to good (in water) the choice of the electrolyte should be based on the extent of stress or the overall response direction for possible actuator or sensor function of the Cell-CNT fiber composite. Similarly, a previous study applied a cellulose MWCNT composite in view of sensing humidity, temperature and piezoresistivity (tensile strain, stress) [40].

**Figure 6:**
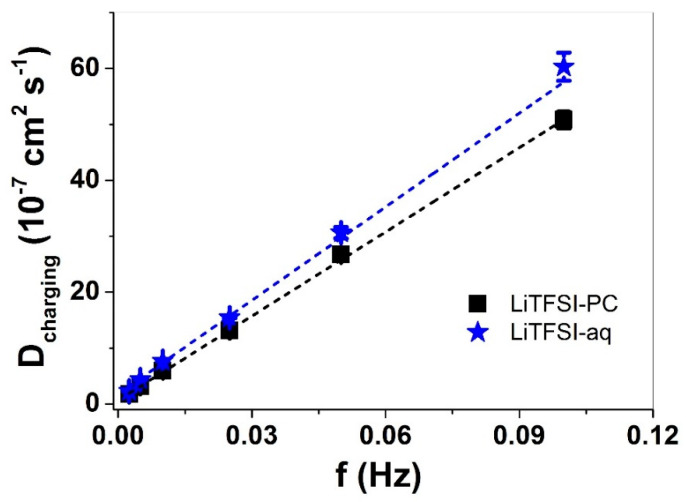
Diffusion coefficients D in Cell-CNT fibers in LiTFSI-PC (■) and LiTFSI-aq (★) electrolytes against frequency upon charging. The linear fits (dashed) are shown here only for orientation.

As the response in water and PC solutions was consistently different, it would be logical to expect a measurable difference also in ion mobility. To prove it, the apparent diffusion coefficients upon charging/discharging were calculated using Equations (1) and (2); the results are shown in Figure 6 (charging) and Figure S6 (discharging).

The diffusion coefficients at charging (Figure 6) and discharging (Figure S6) reveal that with increasing frequency the diffusion coefficient increased linearly. At higher frequency, shorter time is available for ion injection, there is less time for relaxation or parallel processes, and also the amount of charge injected is lower, leading to increased apparent diffusion coefficients. Additional processes are subsided and diffusion coefficients are higher. In the cases of both charging and discharging, the diffusion coefficients in Cell-CNT operated in LiTFSI-aq were 1.2 times higher than in LiTFSI-PC. The lower swelling of cellulose in PC that led to higher stresses clearly limits the diffusion of the ions in the Cell-CNT fibers.

## 4. Conclusions

It can be concluded that in principle it is possible to fabricate linear actuators based on the renewable source of cellulose. As somewhat expected, the Cell-CNT actuation response had a strong solvent dependence. What was not expected, was the nature of the response in propylene carbonate solutions. First, the electromechanical response revealed a change in actuation direction depending on the scan rate in cyclic voltammetry studies. Further investigations by square wave potential step measurements revealed a strong and complex back relaxation process, which decreased with increasing frequency. In the case of LiTFSI-aq, the Cell-CNT fibers behaved like electrochemical capacitors with linear actuation showing expansion upon discharging caused by cation (Li^+^) ingress. The performance of the composite fibers in LiTFSI-aq was far superior than those in LiTFSI-PC; there was 3.4 times higher conductivity leading to 4.3 times higher charge density, reflected in 11 times higher strain. The limited swelling of cellulose in PC was accompanied by 1.2 times lower apparent diffusion coefficients. In both solvents, the actuation remained fairly stable for 100 cycles. Other designs, also with other electrolytes, can be considered in the future to increase the performance in absolute terms; performance was rather limited in the present case.

## Supplementary Materials

The following are available online at https://www.mdpi.com/1996-1944/13/14/3213/s1, Figure S1: Cyclic voltammetric measurements (3rd cycles) of Cell-CNT fibers (working electrode) at different scan rates (5 mV s^−1^ (black); 20 mV s^−1^ (red); 50 mV s^−1^ (green) and 100 mV s^−1^ (blue) operated in three-electrode set up with Ag/AgCl (3M KCl) reference electrode and Platinum sheet counter electrode. The current density j of Cell-CNT in LITFSI-aq is shown in (a) and the charge density Q in (b). Cell-CNT in LiTFSI-PC electrolyte shows current density j in (c) and charge density Q in (d) against the applied potential range 0.55 V to −0.8 V, Figure S2: Square wave current density curves (two subsequent cycles: 2nd and 3rd) at applied frequency 0.005 Hz and potential range 0.55 to −0.8 V (dashed line) of Cell-CNT fibers in LiTFSI-PC (red, dotted) and LiTFSI-aq (black, line) against time t, Figure S3: Square wave potential measurements of Cell-CNT fibers in potential range 0.55 to −0.8 V using different solvents PC (■) and aq (★) with same salt LiTFSI showing in a: stress difference Δσ and in b: strain ε against applied frequencies (logarithmic scale), Figure S4: Linear actuation of stress σ (black line, two subsequent cycles 3rd and 4th) at applied square wave potential measurements of Cell-CNT fibers in potential range 0.55 to −0.8 V (E, dashed line) against time t in LiTFSI-PC at different frequencies of a: 0.01 Hz, b: 0.025 Hz and c: 0.1 Hz, Figure S5: Square wave potential steps (0.1 Hz frequency) of Cell-CNT fibers in LiTFSI-PC (black line) and in LiTFSI-aq (blue line) revealing stress σ against time t, Figure S6: Diffusion coefficients D in Cell-CNT fibers in LiTFSI-PC (■) and LiTFSI-aq (★) electrolytes against frequency upon discharging. The linear fits (dashed) are shown here only for orientation.
