# Supplementary material for: Cellulose-Multiwall Carbon Nanotube Fiber Actuator Behavior in Aqueous and Organic Electrolyte
Source: Materials (Basel). 2020 Jul 19;13(14):3213. doi: 10.3390/ma13143213 (PMC7412319; doi:10.3390/ma13143213)
Supplement: Supplementary file 1 [file materials-13-03213-s001.pdf]

materials

MDPI

Article

Cellulose-multiwall carbon nanotube fiber actuator behavior in aqueous and organic electrolyte

Fred Elhi [1], Anna-Liisa Peikolainen [1], Rudolf Kiefer [2,*] and Tarmo Tamm [1]

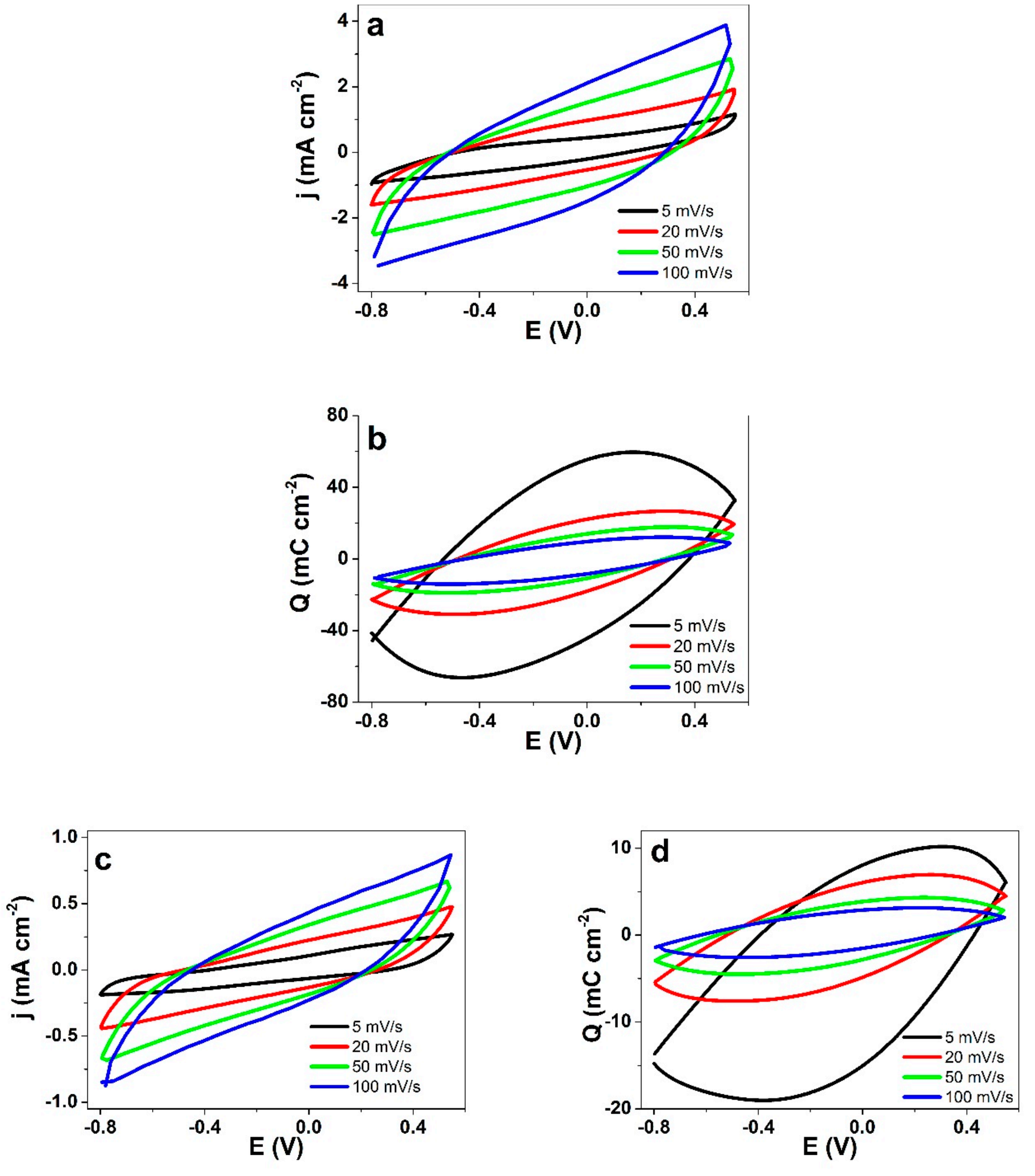

Figure S1. Cyclic voltammetric measurements (3rd cycles) of Cell-CNT fibers (working electrode) at different scan rates (5 mV s^{-1} (black); 20 mV s^{-1} (red); 50 mV s^{-1} (green) and 100 mV s^{-1} (blue) operated in three-electrode set up with Ag/AgCl (3M KCl) reference electrode and Platinum sheet counter electrode. The current density j of Cell-CNT in LITFSI-aq is shown in (a) and the charge density Q in (b). Cell-CNT in LiTFSI-PC electrolyte shows current density j in (c) and charge density Q in (d) against the applied potential range 0.55V to −0.8V.

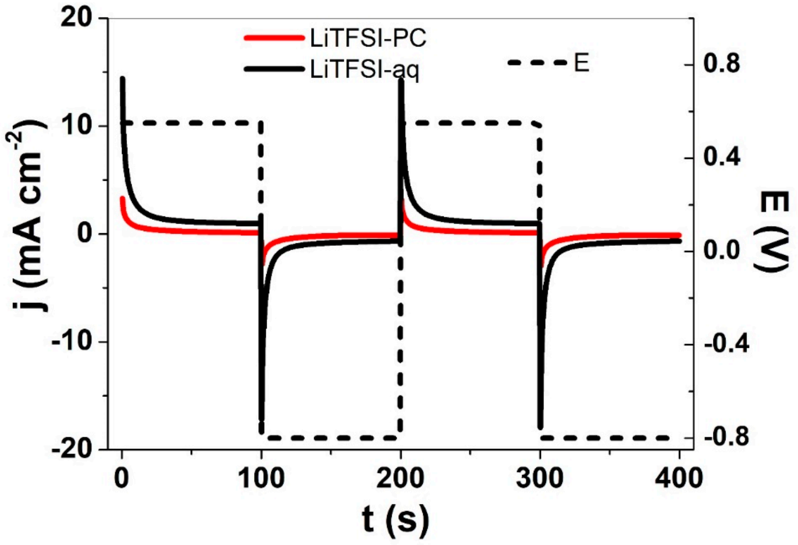

Figure S2. Square wave current density curves (two subsequent cycles: 2nd and 3rd) at applied frequency 0.005 Hz and potential range 0.55 to −0.8 V (dashed line) of Cell-CNT fibers in LiTFSI-PC (red, dotted) and LiTFSI-aq (black, line) against time t.

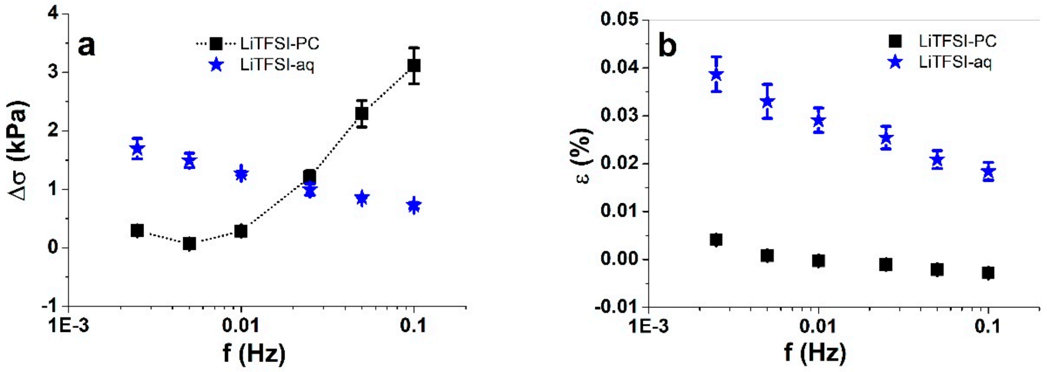

Figure S3. Square wave potential measurements of Cell-CNT fibers in potential range 0.55 to −0.8 V using different solvents PC (■) and aq (★) with same salt LiTFSI showing in a: stress difference Δσ and in b: strain ε against applied frequencies (logarithmic scale).

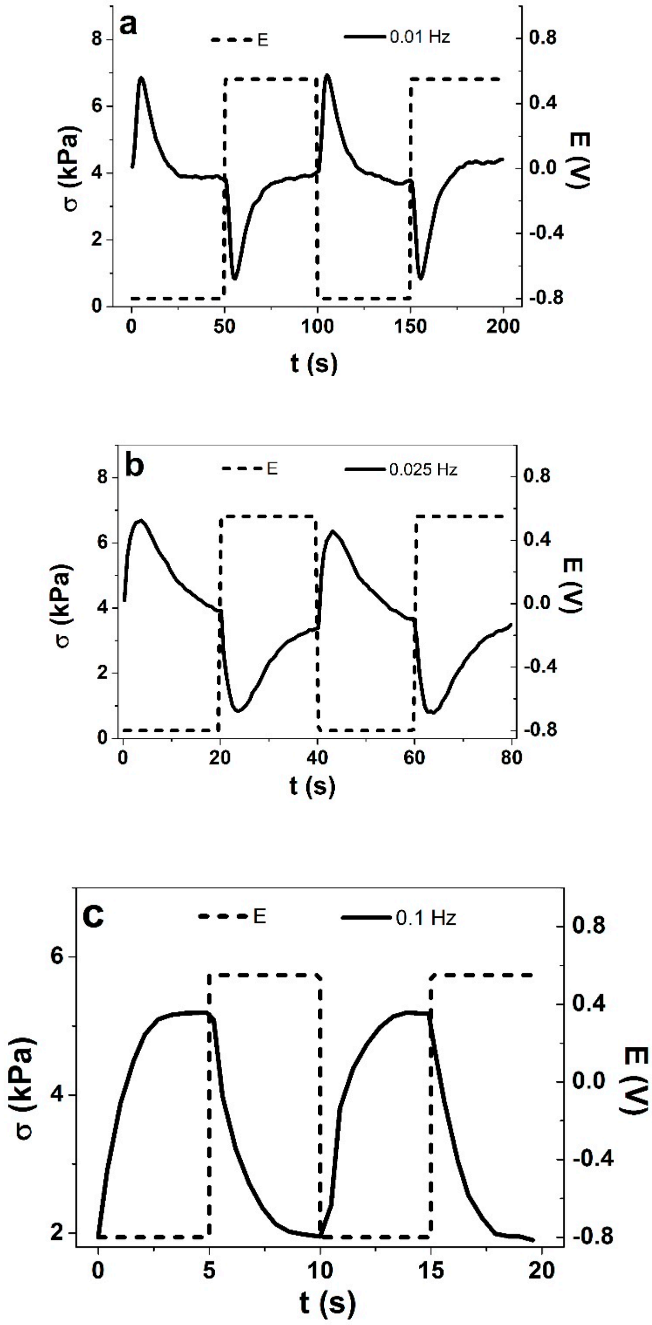

Figure S4. Linear actuation of stress σ (black line, two subsequent cycles 3rd and 4th) at applied square wave potential measurements of Cell-CNT fibers in potential range 0.55 to −0.8 V (E, dashed line) against time t in LiTFSI-PC at different frequencies of a: 0.01 Hz, b: 0.025 Hz and c: 0.1 Hz.

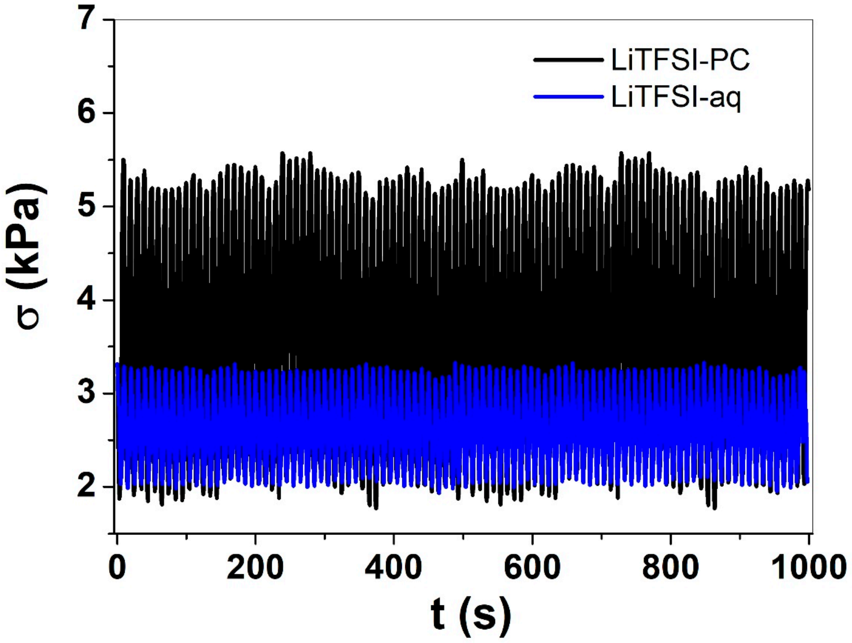

Figure S5. Square wave potential steps (0.1 Hz frequency) of Cell-CNT fibers in LiTFSI-PC (black line) and in LiTFSI-aq (blue line) revealing stress σ against time t.

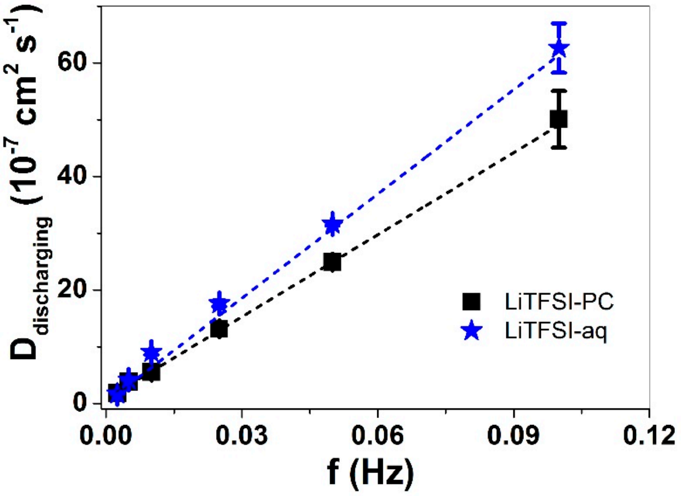

Figure S6. Diffusion coefficients D in Cell-CNT fibers in LiTFSI-PC (■) and LiTFSI-aq (★) electrolytes against frequency upon discharging. The linear fits (dashed) are shown here only for orientation.
